# Optoelectronic properties and strain regulation of the 2D WS_2_/ZnO van der Waals heterostructure

**DOI:** 10.1039/d1ra01877a

**Published:** 2021-04-15

**Authors:** Yujun Guan, Hui Yao, Huahan Zhan, Hao Wang, Yinghui Zhou, Junyong Kang

**Affiliations:** Fujian Provincial Key Laboratory of Semiconductor Materials and Applications, CI Center for OSED, College of Physics Science and Technology, Xiamen University Xiamen 361005 P.R. China huahan@xmu.edu.cn; Research Institute for Biomimetics and Soft Matter, Xiamen University Xiamen 361005 P.R. China

## Abstract

The combination of zinc oxide (ZnO) and transition metal dichalcogenide (TMD) nanoparticles has higher photocatalytic efficiency and field emission performance than TMDs or ZnO, as well as significantly higher water cracking photocatalytic activity. By first-principles calculation, we investigated the structural and optoelectronic properties of the two-dimensional (2D) WS_2_/ZnO van der Waals (vdWs) heterostructure, and the regulation effect of biaxial strain. It is revealed that the conduction-band minimum (CBM) is lower than the reduction potential of water (*E*H^+^_/H2_ ≈ −4.44 eV), and the valence-band maximum (VBM) is lower than the oxidation potential (*E*_O2/H2O_ ≈ −5.67 eV), thus the heterostructure is a good oxidant in the water decomposition process, but cannot match the requirements for water reduction. By applying a −2% biaxial strain, the CBM is elevated to a position higher than the reduction potential of water, then the 2D vdWs WS_2_/ZnO heterostructure becomes a good material for the application of water reduction and other photovoltaic and photocatalytic devices.

## Abstract 2

2D WS_2_/ZnO vdWs heterostructure becomes a good material for water-splitting applications after a strain is applied.

## Graphical abstract

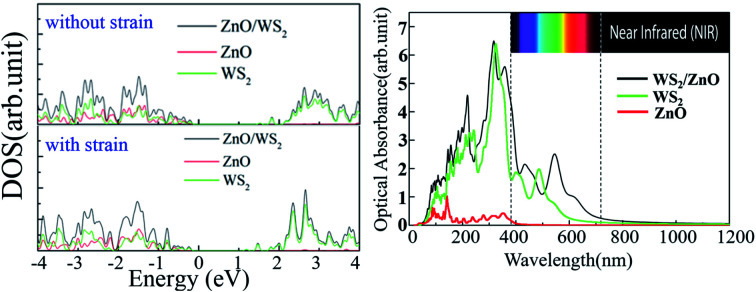

## 1. Introduction

Recently, 2D transition (2D) metal dichalcogenides (MX_2_: M = Mo, W; X = S, Se) have been intensively studied due to their moderate direct bandgap (1.1–2.1 eV), outstanding mechanical characteristics, magneto-optical transport properties, excellent optical properties, and their prospective applications in photovoltaic and photocatalytic devices.^1–5^ However, some materials are prone to recombination of photogenerated electron–hole pairs, and some can only absorb ultraviolet light, leading to the low utilization efficiency of solar energy.

2D van der Waals (vdWs) heterostructures are fabricated by stacking two different 2D materials through weak interactions.^6–8^ These hybrid structures can not only retain the excellent properties of each layer of 2D materials and complement each other, but also provide various methods to regulate the heterostructure such as applying strain to obtain unique structure and performance.^9,10^ A typical vdWs heterostructure usually has a smaller optically active band gap than each component, and therefore exhibits stronger electronic and optical properties than the individual layers. As it is well-known, the conduction-band minimum (CBM) and valence-band maximum (VBM) of type II vdWs heterostructures are located at different layers, therefore it can efficiently separate electronic–hole pairs by transferring the electrons to one layer and the holes to another at the interface, promoting its application prospects in photocatalytic and water decomposition.

Therefore, here we explore the properties and prospect applications of WS_2_/ZnO heterostructure by first principle calculation. Monolayer WS_2_, which can be grown by chemical vapor deposition or be peeled off from bulk WS_2_, exhibits a semiconducting nature with a direct bandgap of 2.1 eV, whereas bulk WS_2_ possesses an indirect bandgap of 1.2 eV.^11^ The bandgap of the bulk WS_2_ bandgap is narrower, and not conducive to the transition and recombination of electron–hole pairs. By contrast, monolayer WS_2_ facilitates visible optical absorption due its relatively narrow and direct bandgap. Many studies focus on the use of monolayer WS_2_ as a potential photocatalyst for splitting water under visible irradiation. However, it suffers from the disadvantage that the photogenerated electron–hole pairs remain in the WS_2_, which results in a high recombination rate. It is clearly necessary to solve this problem to boost its photocatalytic applications.

On the other hand, as a promising direct band gap semiconductor, ZnO has also received extensive attention and been widely studied in related optoelectronic fields.^12,13^ In addition, theoretical simulations show that the band gap of ZnO is very sensitive to the thickness of the layer. As the number of layers decreases, the graphite honeycomb structure is formed in preference to the ZnO (0001) wurtzite structure.^14,15^ Experimental results show that zinc oxide nanosheets with a graphite honeycomb structure have good photocatalytic performance. Nevertheless, due to its wide band gap, monolayer ZnO can only absorb light in the near ultraviolet wavelength range.

Better photoelectrochemical and photocatalytic performance are observed in some composites and heterostructural materials.^16–18^ In recent years, studies have found that the combination of ZnO and transition metal dichalcogenides (TMDs) nanoparticles has higher photocatalytic efficiency and field emission performance than TMDs or ZnO, as well as significantly higher water cracking photocatalytic activity.^19,20^ These results show that 2D WS_2_/ZnO vdWs heterostructure is a promising semiconductor material with excellent properties, which is different from WS_2_ or ZnO monolayers. Our calculation results revealed that the valence-band maximum (VBM) of the WS_2_/ZnO vdWs heterostructure is slightly lower than the oxidation potential of water decomposition. However, peculiar electronic behavior subjected to mechanical strain has been observed in ZnO and some TMDs recently.^21–23^ Thus, a biaxial strain is applied to control the band edge position, regulate the band structure, and make it a good reducing agent in water splitting. At present, 2D MX_2_-based vdWs heterostructures are mainly achieved by transfer techniques.^24,25^ As a side effect, the transfer process usually induce strain in the vdWs heterobilayers. It is also interesting to study whether this strain can induce unique properties in the WS_2_/ZnO vdWs heterostructure.

## 2. Computational procedures

All the calculations in this paper are carried out in Vienna *Ab initio* simulation package (VASP).^26,27^ The Perdew–Burke–Ernzerhof (PBE) correlation functional in the generalized gradient approximation (GGA) is chosen as the exchange correlation potential.^28^ The Projected Augmented Wave (PAW) is used to describe the interaction between valence electrons and ion cores.^29,30^ The model used in the calculation is a 1 × 1 × 1 primitive cell structure. The 2D Brillouin zone is sampled by using the Monkhorst–Pack (MP) scheme with a 13 × 13 × 1 grid centered at *k*-point for structural optimization and 15 × 15 × 1 *k*-points for electronic structure calculations.^31^ A kinetic energy cutoff of 500 eV is utilized for the plane-wave expansion, and the convergence accuracy of force and energy are set to 0.01 eV Å^−1^ and 10^−5^ eV, respectively. In order to more precisely investigate the optoelectronic and structural properties of the system, we adopted the Heyd–Scuseria–Ernzerhof (HSE06) hybrid functional with an accurate exchange ratio of 25%.^32,33^ In addition, we used the DFT-D2 correction method proposed by Grimme *et al.* to describe the vdWs interaction in 2D WS_2_/ZnO heterostructure. At the same time, the vacuum layer along the *Z*-axis is set to 20 Å, which perfectly avoid the interaction between layers due to periodicity.^34,35^

The material under stress will cause a certain deformation of the material's structure, which will further change the electronic properties of the material. There are normal three methods to apply stress: uniaxial stress applied along the *X*-axis (*xx*), uniaxial stress applied along the *Y*-axis (*yy*), and biaxial stress applied along the *X* and *Y* axes (*xy*).^36–38^ In this work, biaxial tensile stress or compressive stress is applied to the heterostructure, and the achieved strain is simulated by changing the lattice parameters of 2D WS_2_/ZnO vdWs heterostructure. The magnitude of the strain is represented by *ε* and is defined as:

1 *ε* = [(*a* − *a*_0_)/*a*_0_] × 100%

where *a* is the lattice constant of 2D WS_2_/ZnO vdWs heterostructure before stress is applied, and *a*_0_ is the lattice constant of the heterostructure after the stress is applied, and a positive value indicates a biaxial tensile strain, a negative value means the application of biaxial compressive strain.

## 3. Results and discussion

### 3.1 Electronic properties of monolayer WS_2_ and monolayer ZnO

**Fig. 1:**
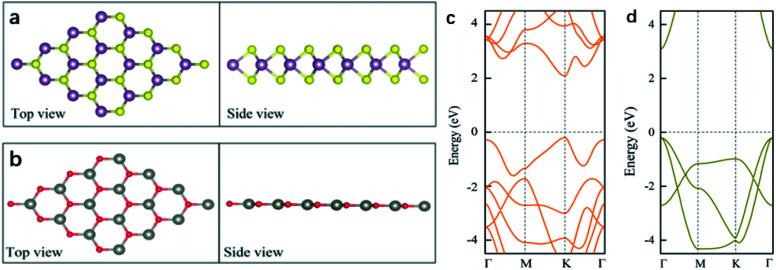
Schematic illustration of crystal structures of (a) pristine 4 × 4 WS_2_ and (b) pristine 4 × 4 ZnO; the band structure of (c) monolayer WS_2_ and (d) monolayer ZnO; red, yellow, grey, and purple spheres represent O, S, Zn, and W atoms, respectively. The Fermi level is set to zero.

Before investigating the 2D WS_2_/ZnO vdWs heterostructure, the structure and electronic properties of monolayer WS_2_ and ZnO were studied. As shown in Fig. 1(a), monolayer WS_2_ presents a graphene-like hexagonal honeycomb configuration and a sandwich structure composed of S–W–S three layers atoms. The lattice constant is *a* = *b* = 3.18 Å, and the bond length of W–S is 2.42 Å. As shown in Fig. 1(b), the ZnO monolayer also has a hexagonal structure, the lattice parameter is *a* = *b* = 3.29 Å, and the Zn–O bond length is 1.90 Å.

Fig. 1(c) and (d) are the energy band diagrams of monolayer WS_2_ and monolayer ZnO, respectively. The Fermi levels in the figure are all at zero eV. According to the Fig. 1(c) and (d), we can draw the following conclusions. Monolayer WS_2_ is a direct band gap semiconductor with both CBM and VBM at the *K* point in the Brillouin zone. Because of this, the material will be more prone to electronic transitions. The calculated bandgap of monolayer WS_2_ is 2.3 eV, whose band edge absorption is located in the visible light region. Monolayer WS_2_ have great potential in the fields of optoelectronic devices and photocatalysis due to these properties. Monolayer ZnO is a semiconductor with a direct bandgap (3.28 eV), which is also a good optoelectronic material with a direct bandgap roughly equivalent to that of bulk ZnO.

**Fig. 2:**
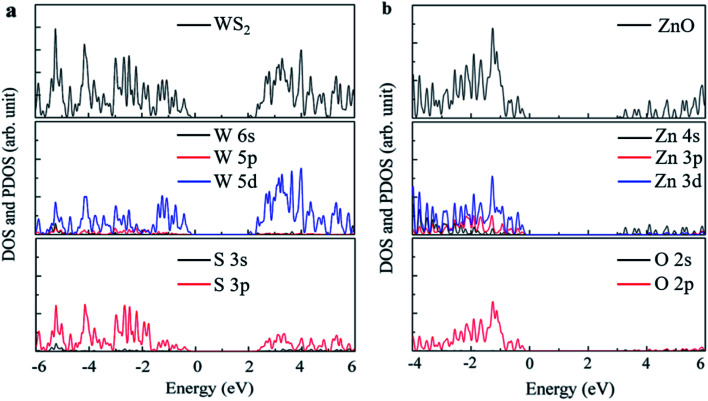
First-principles calculation of monolayer (a) WS_2_ structure and (b) ZnO state density diagram.

The local state density and orbital projection states of monolayer WS_2_ and monolayer ZnO are presented in Fig. 2(a) and (b), respectively. It can be seen that for the monolayer WS_2_, regardless of the conduction band or the valence band, the main contributions are firstly the 5d state of the W atom, and secondly the 3p state of the S atom. In addition, covalence occurs between the 5d state of the W atom and the 3p state of the S atom. Therefore, the monolayer WS_2_ exhibits strong electronic sharing characteristics and excellent mobility within the layer, making it a promising semiconductor optoelectronic material. It can also be seen that the monolayer ZnO has a wide bandgap energy band, the contribution of the conduction band mainly comes from the 4s state and 3d state of Zn, and the contribution of the valence band mainly comes from the 3d state of Zn and the 2p state of O.

### 3.2 Structural properties and stability of 2D WS_2_/ZnO vdWs heterostructure

WS_2_/ZnO vdWs heterostructure can be obtained by superimposing WS_2_ cell and ZnO cell. As shown above, both the monolayer WS_2_ and the monolayer ZnO are direct bandgap semiconductors with bandgaps of 2.30 eV and 3.28 eV, and lattice constants of 3.18 Å and 3.29 Å, respectively. The lattice mismatch between them is small (−3.4%), which is conducive to the construction of a 2D WS_2_/ZnO vdWs heterostructure.

**Fig. 3:**
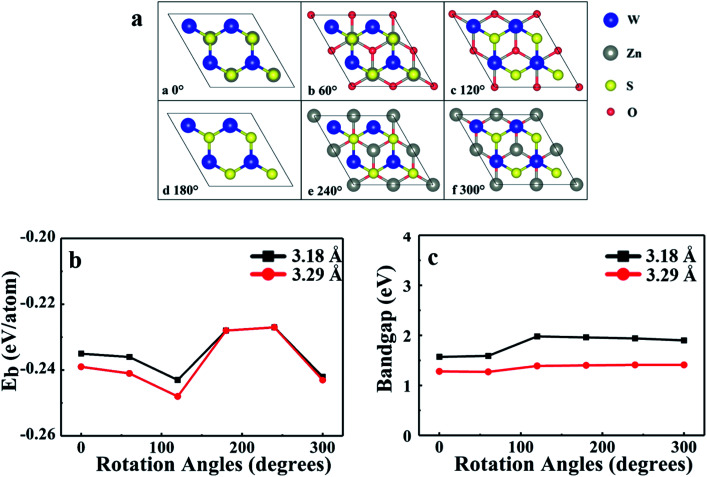
(a) Top views of the six different stacking patterns of 2D WS_2_/ZnO vdWs heterostructure (b) binding energies and (c) bandgaps of 2D WS_2_/ZnO vdWs heterostructure with different rotation angles, the black square and the red dots represents the value when the lattice constant is 3.18 Å and 3.29 Å, respectively.

Taking into account the difference in the initial position of monolayer WS_2_ relative to the monolayer ZnO, a corresponding model can be obtained by fixing the WS_2_ layer and rotating the ZnO layer every 60°, so there are totally six different stacking modes, as shown in Fig. 3(a). Consider the lattice mismatching between the monolayer WS_2_ and the monolayer ZnO, and the stability under different lattice constants, the lattice constants of WS_2_ and ZnO monolayers in heterostructure were both set to 3.18 Å (the same as the monolayer WS_2_), or 3.29 Å (the same as the monolayer ZnO), respectively. By optimizing the structure of these six different stacking modes, the binding energy of each stacking mode is obtained. Fig. 3(b) and (c) show the binding energy and the bandgap of the heterostructure under six different stacking modes and different lattice constants, respectively.

According to the calculation results, we can find that their binding energies are all negative, which indicates that the heterostructure can exist stably. When the rotation angle of monolayer ZnO is 120° (the monolayer WS_2_ is fixed), and the lattice constant of the heterostructure is the same as that of monolayer ZnO, the corresponding stacking mode is the most stable. Therefore, in the subsequent calculations, we choose a 2D WS_2_/ZnO vdWs heterostructure with a lattice constant of 3.29 Å and a ZnO rotation angle of 120°. In the heterostructure, regardless of the stacking mode, the Zn–O bond length, which always is 1.90 Å, does not change. It is easy to understand, because the lattice constant of the heterostructure is consistent with the lattice constant of monolayer ZnO. In the six stacked heterostructures modes corresponding to the ZnO rotation angle from 0 to 300°, the W–S bond lengths are about 2.44 Å, slightly longer than the bond length of monolayer WS_2_ (2.42 Å), which is caused by lattice mismatch. The stability of WS_2_/ZnO vdWs heterostructure with different stacking angles will be experimentally investigated when the monolayer ZnO is fabricated.

### 3.3 Electronic properties of 2D WS_2_/ZnO vdWs heterostructure

To investigate the electronic properties of monolayer WS_2_ and monolayer ZnO after the formation of the vdWs heterostructure, we calculated the band structure of the optimized 2D WS_2_/ZnO vdWs heterostructure. Fig. 3(c) shows the band gap change of the WS_2_/ZnO heterostructure mode corresponding to different ZnO rotation angles. When the heterostructure lattice constant is 3.29 Å, the band gap values corresponding to the ZnO rotation angle from zero to 300° are 1.28 eV, 1.27 eV, 1.39 eV, 1.40 eV, 1.41 eV, and 1.41 eV. When the heterostructure lattice constant is 3.18 Å, the band gap values of the model corresponding to the rotation angle from zero to 300° are 1.57 eV, 1.59 eV, 1.98 eV, 1.96 eV, 1.94 eV and 1.90 eV. Compared with the band gap of monolayer ZnO (3.28 eV), the band gap of the WS_2_/ZnO heterostructure is much narrower. This implies that 2D WS_2_/ZnO vdWs heterostructure is beneficial to the absorption of visible light. In addition, the calculation results also show that when the ZnO rotation angle changes, the band gap of 2D WS_2_/ZnO vdWs heterostructure remains almost unchanged, which is wider than the required bandgap. Therefore, the strain can regulate the water splitting photocatalytic performance, making the 2D WS_2_/ZnO vdWs heterostructure a good application prospect in the field of photocatalysis. Potential of water, which can be used as an oxidant in water splitting.

**Fig. 4:**
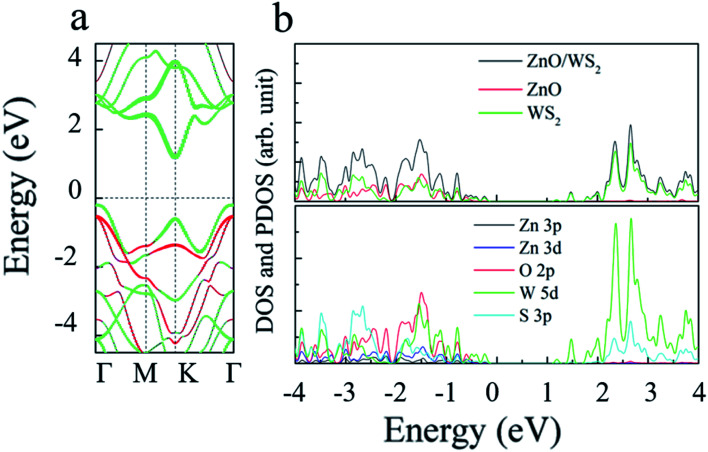
(a) The band structure; red and green curves represent the contribution of ZnO and WS_2_, respectively; (b) density of states of the 2D WS_2_/ZnO vdWs heterostructure with the rotation angles of 120°.

Which also means that the ZnO rotation angle of the heterostructure has little effect on the band gap. In another sentence, the impact of ZnO rotation angle is negligible, and different stacking modes will not affect our conclusions in terms of quality. The band diagram of 2D WS_2_/ZnO vdWs heterostructure with a lattice constant of 3.29 Å and a rotation angle of 120° is shown in Fig. 4(a). It can be seen that the heterostructure is an indirect band gap semiconductor. Regardless of VB and CB, energy levels near Fermi level mainly associated with the WS_2_ layer (the blue part in the figure). In detail, Fig. 4(b) shows the local density of states and orbital projection of 2D WS_2_/ZnO vdWs heterostructure. It can be seen that the contribution of the conduction band of the heterostructure comes from the 5d orbital of the W atom and the 3p orbital of the S atom. The main contribution of the valence band comes from the 5d orbital of the W atom. The ZnO layer also has a small part of the contribution, coming from the 2p orbital of the O atom. This further shows that compared with monolayer WS_2_ and monolayer ZnO, the electron–hole pairs in the heterostructure in CBM and VBM are more likely to be spatially separated.

**Fig. 5:**
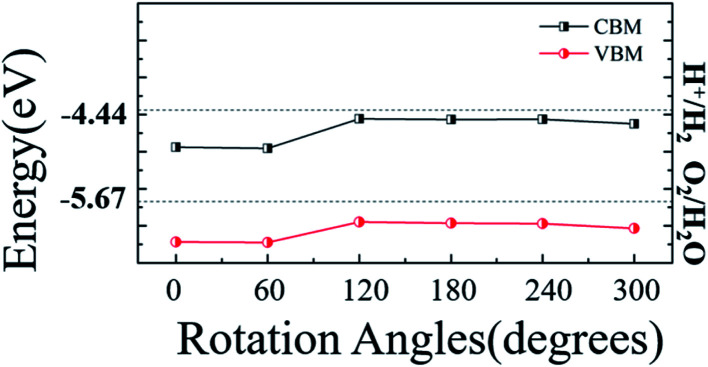
The band alignment of the 2D WS_2_/ZnO vdWs heterostructure of different rotation angles with respect to the water redox levels.

To explore the application of WS_2_/ZnO heterostructure in water splitting, photovoltaic and photocatalytic devices, its band edges alignment with respect to the redox potential of water is shown in Fig. 5, band edges of other stacking modes under different rotation angles are also presented as a reference. These calculation results show that the CBM of any stack mode is lower than the reduction potential of water (*E*H^+^_/H_2__, about −4.44 eV), and VBM is also lower than the oxidation potential (*E*_O_2_/H_2_O_, about it is −5.67 eV),^39,40^ and when the rotation angle is 120°, the VBM is slightly close to the oxidation potential of water. This means in the water splitting process that 2D WS_2_/ZnO vdWs heterostructure material can be used as a good oxidant, but cannot be used as a good reducing agent. This hinders its application in photocatalytic water splitting. To solve this problem, we apply a strain to regulate the band edge position of the heterostructure, to see if the 2D WS_2_/ZnO vdWs heterostructure material become a good oxidation in the water decomposition reducing agent.

### 3.4 Strain regulation of 2D WS_2_/ZnO vdWs heterostructure electronic structure

**Fig. 6:**
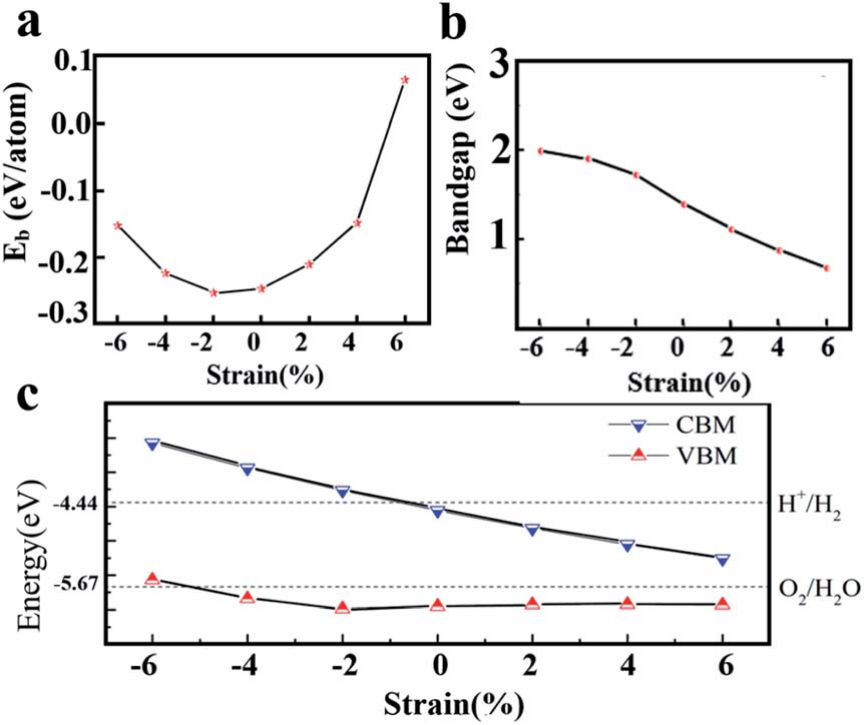
(a) Banding energies and (b) bandgaps of 2D WS_2_/ZnO vdWs heterostructure with different strains (c) the band alignment of the 2D WS_2_/ZnO vdWs heterostructure of different strains with respect to the water redox levels.

When two materials with different lattice constants form a heterostructure, the strain can affect the crystal structure and electronic properties of the material. In addition, many studies shown that the electronic and optical properties of 2D materials can be effectively regulated by applying strain. The binding energy and bandgap changes of heterostructure under different strains are shown in Fig. 6(a) and (b). The model used here is the 2D WS_2_/ZnO vdWs heterostructure corresponding to a lattice constant of 3.29 Å and a rotation angle of 120°. The results show that the binding energy decreases when the strain changes from −6% to −2%, and increases when the strain changes from −2% to +6%, which means that the 2D WS_2_/ZnO vdWs heterostructure is a more stable configuration when the −2% strain (compression) is applied to the initial heterostructure. Moreover, in the range of strain from −6% to +6%, the bandgap values of heterostructure are 1.99 eV, 1.91 eV, 1.72 eV, 1.39 eV, 1.11 eV, 0.87 eV and 0.67 eV, respectively.

The band alignment diagram of 2D WS_2_/ZnO vdWs heterostructure with respect to the water redox potential under different strains is shown in Fig. 6(c). It shows that the strain can adjust the band edge position of the heterostructure. For the CBM, when the strain is between −6% and −2%, the CBM is higher than the reduction potential of water, which can be used as a reducing agent in water decomposition, when the strain is between −2% and +6%. The CBM gradually decreases and is lower than the reduction potential of water, and cannot be used as a reducing agent in water decomposition. For the VBM, when the strain is between +6% and −4%, the VBM is still lower than the oxidation potential of water, which can be used as an oxidant in water splitting.

For the application in water splitting, the VBM should be lower than the oxidation potential of water (*E*_O_2_/H_2_O_, about −5.67 eV), and the CBM should be higher than the reduction potential of water (*E*H^+^_/H_2__, about −4.44 eV), requiring a bandgap wider or equal to 1.23 eV. As is shown in Fig. 6(b), under a −2% strain, the band gap of WS_2_/ZnO heterostructure is 1.79 eV, which is wider than the required bandgap. Therefore, the strain can regulate the water splitting photocatalytic performance, making the 2D WS_2_/ZnO vdWs heterostructure a good application prospect in the field of photocatalysis.

**Fig. 7:**
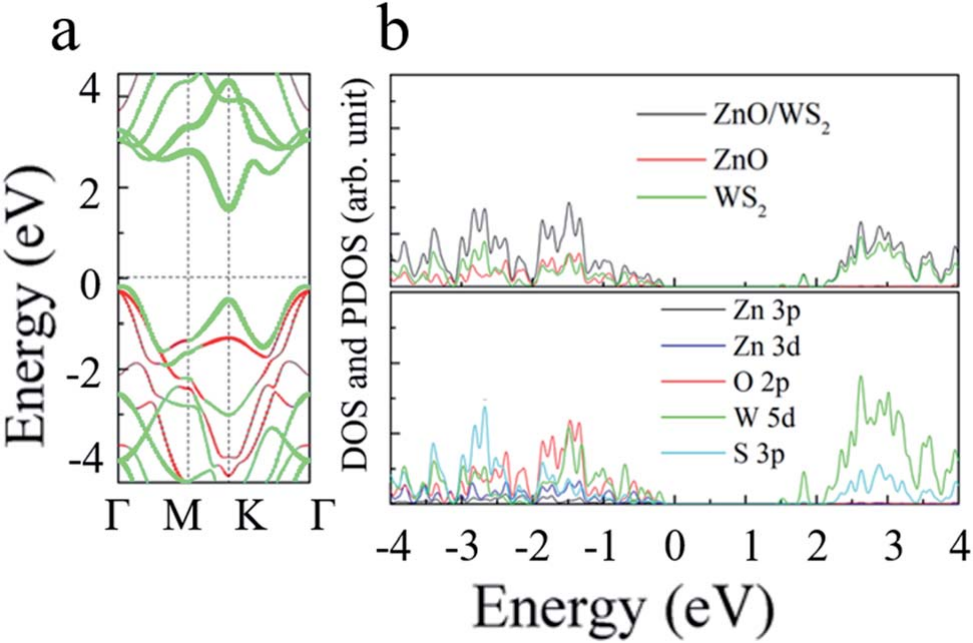
(a) The band structure; red and green curves represent the contribution of ZnO and WS_2_, respectively; (b) density of states of the 2D WS_2_/ZnO vdWs heterostructure with the −2% strain.

**Fig. 8:**
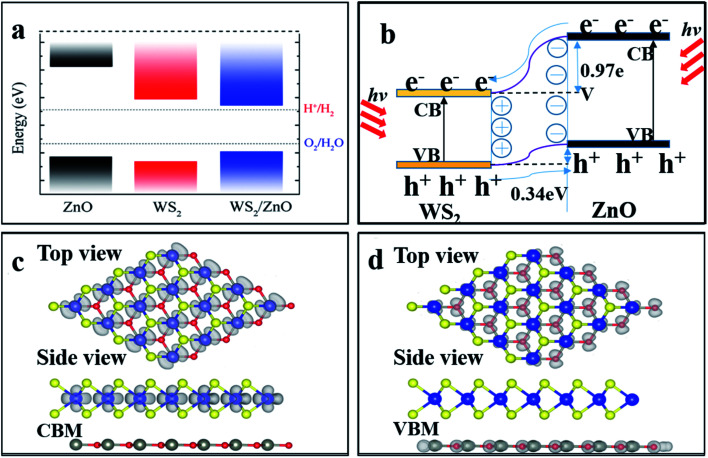
(a) Band edges of monolayer WS_2_, ZnO, and 2D WS_2_/ZnO vdWs heterostructure. The dashed lines indicate the energy levels of redox potentials of H^+^/H_2_ (−4.44 eV) and O_2_/H_2_O (−5.67 eV). (b) Transfer and separation of photogenerated electron–hole pairs at the 2D WS_2_/ZnO vdWs heterostructure interface. Top view and side view of partial charge densities of (c) CBM and (d) VBM of 2D WS_2_/ZnO vdWs heterostructure, the value of the isosurface is set to 0.03 eÅ^−3^.

The energy band, local density of states and orbital projection density of states of 2D WS_2_/ZnO vdWs heterostructure under −2% strain are shown in Fig. 7(a) and (b). It can be seen that the heterostructure is still an indirect bandgap material. The conduction band is mainly contributed by the 5d orbitals of W atoms and the 3p orbitals of S atoms. The valence band is mainly contributed by the 5d orbital of W atoms, and a small part of the 2p orbital of O atoms. Considering the electronic transitions selection rules, the electrons are mainly excited from the orbitals of 5d of the W atom, 2p of the O atom and 3p of the S atom, to the orbitals of 3p of the S atom, and 5d of the W atom. The band alignment diagram of monolayer WS_2_, monolayer ZnO and 2D WS_2_/ZnO vdWs heterostructure shows that the vdWs heterostructure is typical type-II heterostructure, as shown in Fig. 8(a). In order to further demonstrate that 2D WS_2_/ZnO vdWs heterostructure with type-II can effectively reduce the recombination of photogenerated carriers, we calculated the VBM and CBM offsets (Δ*E*_V_ and Δ*E*_c_) between the WS_2_ monolayer and the ZnO monolayer in the heterostructure, which are approximately 0.34 eV and 0.97 eV, respectively, as shown in Fig. 8(b). When it is irradiated, the holes in the valence band of the WS_2_ layer move toward the valence band of the ZnO layer, and the electrons in the conduction of the ZnO layer move toward the conduction band of the WS_2_ layer.

The valence band of the ZnO layer, and the photogenerated electrons transfer from the conduction band of the ZnO layer to the conduction band of the WS_2_ layer. Therefore, the VBM and CBM offsets (Δ*E*_V_ and Δ*E*_c_) indicate that the photo-generated carries in the type-II heterostructure material tend to form a separation in space, reducing the recombination rate of photo-generated carriers. Fig. 8(c) and (d) show the partial charge density at CBM and VBM of 2D WS_2_/ZnO vdWs heterostructure. The charge at CBM is concentrated at the monolayer WS_2_ and the charge at VBM is concentrated at the monolayer ZnO. This further prove that the band edges offsets help to effectively separate the photogenerated electrons and holes at the interface of 2D WS_2_/ZnO vdWs heterostructure.

**Fig. 9:**
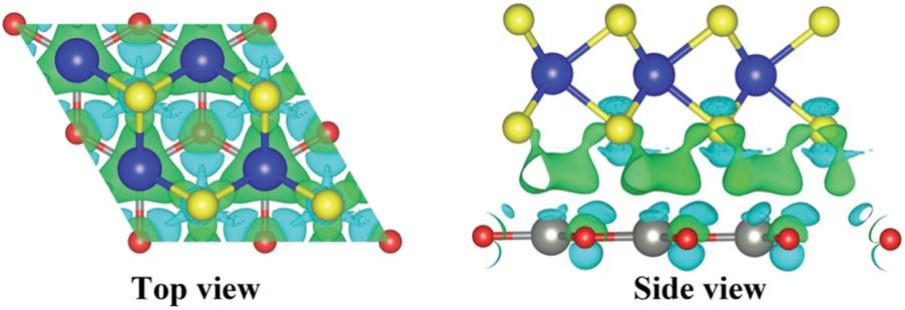
(a) Top and (b) side views of charge density difference of the 2D WS_2_/ZnO vdWs heterostructure, cyan and green regions represent charge depletion and accumulation, respectively.

In order to explore the charge transfer between layers after the formation of 2D WS_2_/ZnO heterostructure, we use the following formula to calculate the differential charge density of the system:

2 Δ*ρ* = *ρ*_WS_2_/ZnO_ − *ρ*_ZnO_ − *ρ*_WS_2__

where *ρ*_WS_2_/ZnO_, *ρ*_ZnO_ and *ρ*_WS_2__ represent 2D WS_2_/ZnO vdWs heterostructure, the charge density of monolayer ZnO and monolayer WS_2_, as shown in Fig. 9(a) and (b), where the cyan area indicates a decrease in electrons, and the green area indicates an increase in electrons. Due to the weak vdWs interaction between the layers, the charge transfer mainly occurs in the ZnO layer and the S atomic layer adjacent to ZnO. Because of the redistribution of charge at the interface, the effective separation of electron–hole pairs in 2D WS_2_/ZnO vdWs heterostructure can be attributed to the formation of electric dipoles.^41^

### 3.5 Optical properties of two-dimensional WS_2_/ZnO vdWs heterostructure

As we all know, most of the wavelengths of light incident on the earth are in the near-infrared and visible light range. Generally speaking, as a high efficient photocatalytic material, the light absorption ability is closely related to its electronic structure, and has an important impact on its photocatalytic activity.

**Fig. 10:**
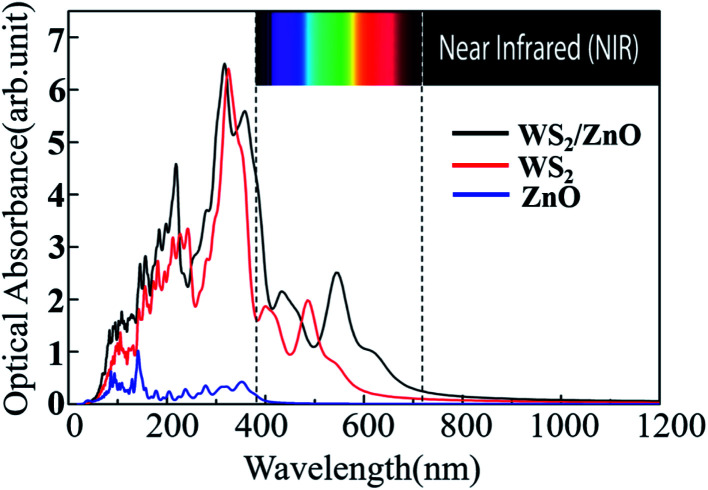
Optical absorption curves of monolayer WS_2_, monolayer ZnO, and 2D WS_2_/ZnO vdWs heterostructure.

We calculated the imaginary part of the dielectric function of monolayer WS_2_, monolayer ZnO, and two-dimensional WS_2_/ZnO vdWs heterostructures to obtain the corresponding light absorption curves, as shown in Fig. 10. Compared with the monolayer WS_2_, the 2D WS_2_/ZnO vdWs heterostructure broadens the visible light absorption range, and the two peaks in the visible light region are red-shifted (397.56 nm to 433.75 nm, 487.50 nm to 548.75 nm), while the monolayer ZnO only has a wide band gap, responding to the near-ultraviolet light region. The 2D WS_2_/ZnO vdWs heterostructure not only responds to light in the near-ultraviolet region, but also responds to light in the visible light region, therefore broadening the light absorption wavelength range of the material. Therefore, the 2D WS_2_/ZnO vdWs heterostructure is more effective in the utilization of solar energy, which improves the photocatalytic performance, and can be applied to photovoltaic and photocatalytic devices.

## 4. Conclusions

Herein we use the VASP to study the geometric structure, electronic properties, band edge alignment, differential charge density and photoelectric properties of the 2D WS_2_/ZnO vdWs heterostructure. Among the six possible stacking configuration modes, we found that when the lattice constant is 3.29 Å and the rotation angle is 120°, the heterostructure is the most stable with the lowest binding energy. The band structure and charge density distribution show that the 2D WS_2_/ZnO vdWs heterostructure has a wider range of light absorption, which is an inherent type II characteristic. No matter which configuration mode of the heterostructure, the CBM is lower than the reduction potential of water (*E*H^+^_/H_2__, about −4.44 eV), and the VBM is slightly lower than the oxidation potential of water (*E*O__2_/H_2_O_, about −5.67 eV), showing that the 2D WS_2_/ZnO vdWs heterostructure can be a good oxidant in the water decomposition process, but cannot match the requirements for water reduction. However, after a −2% strain is applied, the CBM shifts from the original reduction potential lower than that of water to higher than the reduction potential of water, thus overcoming its disadvantage and making the 2D WS_2_/ZnO vdWs heterostructure a good material for water-splitting applications in photocatalysis and other fields.

## Conflicts of interest

There are no conflicts to declare.

## Supplementary Material
